# The mitral to aortic/pulmonary velocity-time integral ratio is a simple, feasible and accurate discriminator for echocardiographic evaluation of severe isolated mitral regurgitation

**DOI:** 10.1007/s10554-024-03249-x

**Published:** 2024-10-17

**Authors:** Nitesh Nerlekar, Satish Ramkumar, Paul Maggiore, Justin Teng, Cengiz Cimenkaya, Kim Kuy Be, Angus Baumann, Stephen J. Nicholls, Stuart Moir

**Affiliations:** 1 Monash Cardiovascular Research Centre, Monash University and Victorian Heart Hospital, Monash Health, Clayton, VIC Australia; 2 Baker Heart and Diabetes Institute, Melbourne, VIC Australia

**Keywords:** Mitral valve insufficiency, Echocardiography, Doppler, Pulsed, MAVIR, Mitral-aortic velocity-time integral ratio, MPVIR, Mitral-pulmonary velocity-time integral ratio

## Abstract

Echocardiographic quantification of mitral regurgitation (MR) remains challenging, requiring dedicated image acquisition, and is limited by potential error from geometric assumptions of annular dimensions. Volume is a product of area and flow and assuming proportional mitral/aortic areas, an increased mitral-inflow volume compared to LV/RV-outflow semi-quantitatively represents greater MR regurgitant volume. Therefore, we investigated the feasibility and diagnostic performance of the mitral-aortic velocity-time integral(VTI) ratio in isolated MR. We also investigated the use of the mitral-pulmonary VTI ratio as an alternative in clinical situations where the LV outflow tract(LVOT) VTI could not be used. We reviewed 166 consecutive patients (33%, *n* = 54 severe MR by multi-parameter integrated expert opinion). Pulsed wave Doppler VTI at the mitral leaflet tips and the left ventricular outflow and continuous-wave Doppler of the RV outflow tract were measured individually and independently by blinded readers(expert and trainee status) to derive the ratio. Receiver operator characteristic area under the curve(AUC) comparison was calculated and compared with effective regurgitant orifice area(EROA > 40 mm), regurgitant volume(RVol > 60mL), vena contracta(VC > 0.7 cm), E-velocity > 1.2 cm, systolic flow reversal(SFR), left atrial and ventricular dilatation. Increasing ratio was associated with severe MR(AUC 0.94) with optimal threshold defined at 1.3. This provided significant discrimination for severe MR(AUC 0.81) compared to EROA(0.68), VC(0.52), LV dilatation(0.69), LA dilatation(0.70), SFR(0.73), E-velocity(0.68) all *p* < 0.05, with sensitivity 82% and specificity 94%. The mitral-pulmonary VTI ratio demonstrated similar discrimination(AUC 0.92) with optimal threshold defined at 1.14. Excellent inter-observer reproducibility(intra-class correlation 0.97) was seen between trainee and expert readers. There was no difference in AUC comparison by MR mechanism or patient rhythm. The mitral-aortic and mitral-pulmonary VTI ratios are simple, geometric-free parameters feasibly reproducible from routine echocardiographic datasets and are excellent discriminative tools for severe MR. Readers should consider integration of this parameter in routine reporting.

## Introduction

The echocardiographic assessment of mitral regurgitation (MR) assessment remains challenging with multiple proposed methods involving both qualitative and quantitative metrics. No single marker has been accepted to provide adequate discrimination of severe mitral regurgitation and accordingly, the latest iteration of guidelines for MR assessment recommends an integrative approach using transthoracic echocardiography [1]. Concordance between echocardiographic methods is also poor [2], often require considerable operator and interpreter experience and ideally a more simplified metric to distinguish severe MR is clinically desirable. The parameters considered to be most effective or concordant, are quantitative measures employing the principle of mass conservation using the proximal isovelocity surface area (PISA technique) [2]. However, a significant drawback of this technique, and indeed several quantitative echocardiographic methods is the need for a dedicated acquisition, and the error associated with geometric assumptions. PISA measurements assume a hemispheric shell at the point of flow convergence, annular dimensions assume circular geometry and subsequently errors are squared, and the downstream complexity of equations the incorporate several of these variables may introduce multiplicative margins of error [3].

Quantification of regurgitant severity based on comparison of stroke volumes across the mitral annulus and LV outflow tract (LVOT) is an established alternative [4], but this technique (where stroke volume is the product of VTI and cross-sectional area), is plagued by the geometric assumptions of converting linear annular diameters into cross sectional area for calculation of stroke volume, with the non-circular, saddle shaped mitral annulus posing considerable difficulty.

In the mid 1990’s Triboulloy et al. demonstrated that the ‘dimensionless’ pulsed-wave Doppler VTI ratio of the mitral inflow (acquired at the mitral leaflet tips) to the LVOT VTI approximates 1 in normal subjects and that and that increasing ratios greater than 1 could be utilised as a non-geometric index for semiquantitative assessment of mitral regurgitation compared to angiographic severity [5]. The use of this metric has not been widely adopted, and evaluation against more contemporary echocardiographic parameters has not been adequately described.

The aim of this study was to evaluate the efficacy of the mitral-aortic VTI ratio in discriminating severe isolated mitral regurgitation when compared with contemporary echo approaches. We also sought to compare the mitral-pulmonary VTI ratio to the mitral-aortic VTI ratio as an alternative in clinical situations where the LVOT VTI could not be used.

## Methods

### Population

This retrospective study was performed at a high-volume quaternary imaging centre and the reference standard for mitral regurgitation severity was determined by the estimation of expert cardiologists with formalised subspecialty training in echocardiography, and the determination of mitral regurgitation severity based on clinical and echocardiographic integrative parameters as per the latest guidelines [1, 4]. Approval was obtained from the institution’s Human Research Ethics Committee. Patients who were defined as having severe mitral regurgitation were identified from the Monash Heart echocardiography repository and comprised patients formally adjudicated by the Heart Team prior to a decision on intervention in a multi-disciplinary setting between 2014 and 2018 and we studied a subset with isolated non-rheumatic mitral regurgitation, no significant annular calcification and no more than mild aortic regurgitation. Patients with pulmonary valve dysfunction (stenosis or regurgitation), moderate or greater tricuspid regurgitation were also excluded. A group of patients deemed to have non-severe isolated mitral regurgitation (grade1-3) (without MAC or AR), or no valvular disease (grade 0) were included as a comparator group.

### Echocardiographic analysis

Echocardiograms were performed on Philips IE33 or GE machines. Where possible, ASE recommended parameters for mitral regurgitation severity were evaluated during the echo, or during post-processing [4]. This included: valve morphology and MR mechanism, left ventricular (LV) cavity dimension at end-diastole indexed to body surface area, estimated LV ejection fraction, left atrial volume indexed to body surface area, multiple view assessment of the mitral regurgitation jet with colour Doppler, pulmonary venous flow pattern (SFR), estimation of pulmonary artery systolic pressure, venae contracta width, proximal isovelocity surface area (PISA) radius and continuous wave Doppler of MR jet to allow for effective regurgitant orifice area (EROA) and regurgitant volume calculation. Blood pressure, heart rate and rhythm were all obtained. The mitral-aortic VTI ratio was obtained with pulsed Doppler at the mitral leaflet tips in the apical 4-chamber view and at the LV outflow tract (LVOT) in the apical 3-chamber view. The average value of three cardiac cycles was used, and in the case of atrial fibrillation or pacing, the average value of five cardiac cycles was used.

We also calculated the mitral-pulmonary VTI ratio as an alternative to the mitral-aortic ratio. Pulmonary flow is equivalent to aortic flow in the absence of pulmonary valve regurgitation or intra-cardiac shunts. This is clinically useful in situations where the LVOT VTI is unable to be used accurately such as the presence of aortic regurgitation or LVOT obstruction. Continuous wave Doppler across the RVOT was performed in the parasternal short axis view and the VTI measured (average of three cardiac cycles). The RVOT pulsed wave Doppler VTI was not used as there is often difficulty in clinical practice in identifying the pulmonary valve leaflets, leading to inaccuracy. The VTI of the continuous wave Doppler signal was felt to be a close approximation to the RVOT pulsed wave VTI (in the absence of pulmonary valve dysfunction).

All echocardiograms were de-identified and reviewers blinded to patient data. Expert cardiology reviewers with established echocardiographic experience analysed the studies as well as fellows-in-training with an overlap of different measurement parameters to identify inter-observer variability. To further minimise bias, assessment of Doppler parameters was performed *individually and independently* to ensure that the reviewing cardiologist did not analyse both LVOT and MR Doppler parameters in the same patient. Similarly, independent assessment for mitral regurgitation qualitative and quantitative parameters was also performed.

### Statistical analysis

Categorical variables are described as numbers and percentages and analysed with the chi-square tested. Continuous variables are described as mean with standard deviation and analysed using a t-test. Inter and intra-observer variability was measured with the intra-class correlation coefficient. Logistic regression, using severe mitral regurgitation as the outcome was performed for each echocardiographic variable. Variables such as vena contracta width and the mitral-aortic ratio which are non-integer values were multiplied by a factor of 10 (to reflect a 0.1 unit increase in each parameter) to incorporate into models given the known expectation of a change in log odds of the outcome reflective of a change in 1 unit of the independent variable. Multivariable logistic regression analysis was not performed due to collinearity amongst echo parameters. Receiver operator curve analysis with area under the curve was calculated for each metric and results combined using the method of DeLong et al. The net reclassification index was performed to evaluate the proportion of reclassified MR patients compared to using a model of EROA and vena contracta width. Regurgitant volume and EROA were not included in the same models given their collinearity.

## Results

**Fig. 1:**
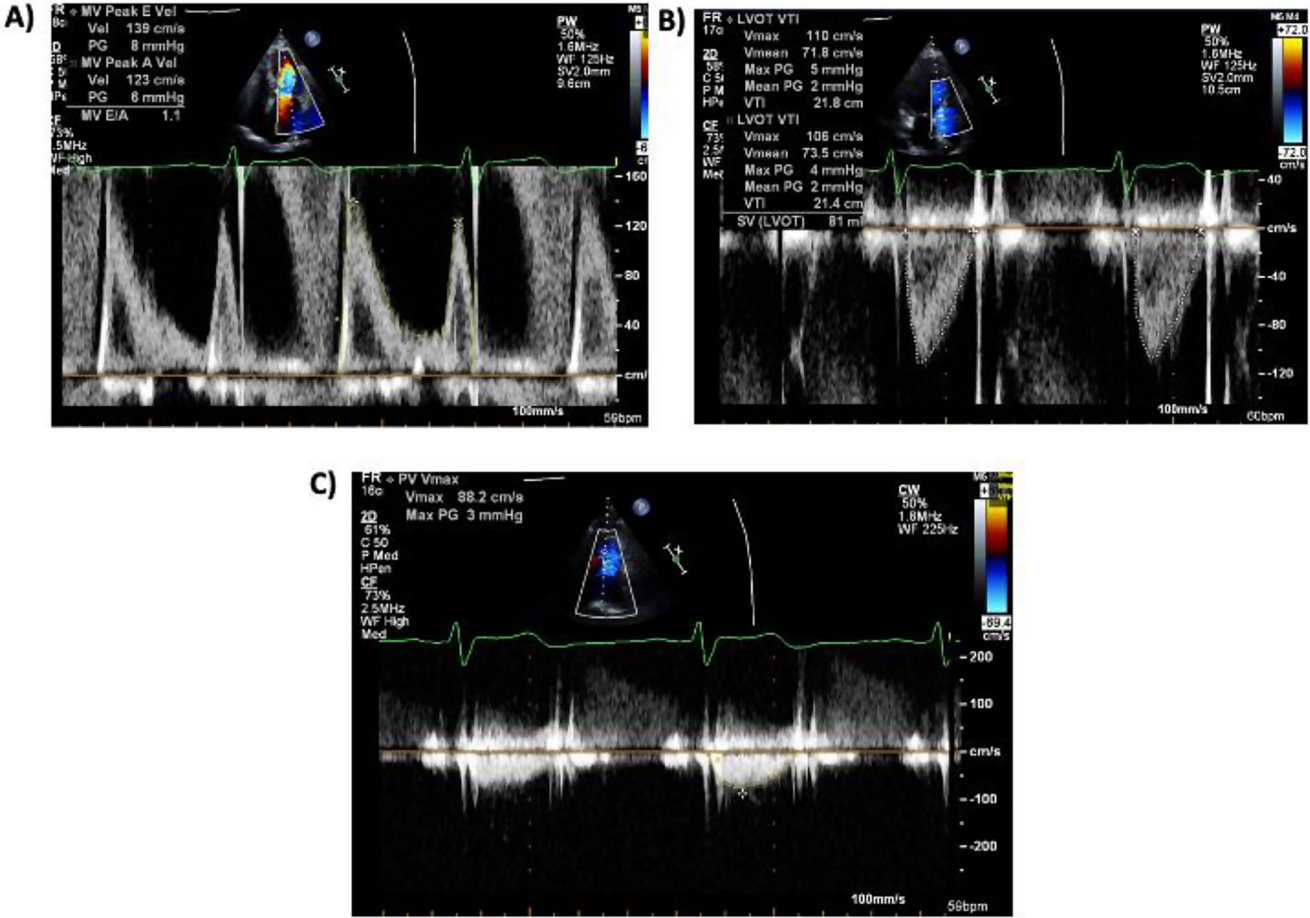
Example of the measurement of the mitral valve VTI, LVOT VTI and RVOT Continuous Wave Doppler VTI in a patient with severe mitral regurgitation. Panel **A** (top left) shows the calculation of the MV VTI which is performed by pulse wave Doppler at the leaflet tips in the apical 4 chamber view (trace in yellow outline). Panel **B** (top right) shows the calculation of the LVOT VTI performed by pulse wave Doppler of the LVOT in the apical 3 chamber view (trace in white outline). The is also measured in the apical 5 chamber view and the results averaged. Panel **C** (bottom) shows the calculation of the RVOT VTI which is performed by continuous wave Doppler of the RVOT in the parasternal short axis view (trace in yellow outline). The MV VTI: LVOT VTI Ratio is 41/21.6 = 1.9. The MV VTI: RVOT VTI Ratio is 41/17.4 = 2.4

There were 166 included patients (59% male, *n* = 98), comprising 54 (33%) with severe (grade 4) mitral regurgitation and 112 comparator patients comprising 63 (38%) with no (grade 0) regurgitation and 49 (29%) with grade 1–3 mitral regurgitation. Patients with severe MR were older (69.7 vs. 58.6 years; *p* < 0.001) and there was a high proportion of male patients (70.1% vs. 53.6%; *p* = 0.039). Where the cause of MR was clearly evaluable, 39 had primary MR and 37 had secondary MR. Baseline parameter comparisons in each group are demonstrated in Table 1. An example of the measurement of the mitral-aortic and mitral-pulmonary VTI ratio is demonstrated in Fig. 1.

**Table 1:** Baseline parameters by MR Severity

Parameter	Severe MR	Non-severe MR	*p*-value
MV VTI: LVOT VTI Ratio (*n* = 166)	1.65 ± 0.48 (*n* = 54)	0.99 ± 0.24 (*n* = 112)	< 0.001
MV VTI: RVOT CW Ratio (*n* = 162)	1.69 ± 0.54 (*n* = 51)	0.97 ± 0.26 (*n* = 111)	< 0.001
Vena contracta (*n* = 96) (cm)	0.50 ± 0.18 (*n* = 53)	0.30 ± 0.19 (*n* = 43)	< 0.001
EROA (*n* = 85) (cm^2^)	52.4 ± 42.7 (*n* = 51)	21.6 ± 19.4 (*n* = 34)	< 0.001
RVol (*n* = 80) (mL)	66.9 ± 54.1 (*n* = 50)	29.4 ± 16.9 (*n* = 30)	< 0.001
Ejection Fraction (*n* = 166) (%)	46.6 ± 19.6 (*n* = 54)	54.6 ± 13.2 (*n* = 112)	0.002
E-velocity (*n* = 163) (m/sec)	1.19 ± 0.28 (*n* = 52)	0.83 ± 0.44 (*n* = 111)	< 0.001
LA Volume (*n* = 161) (mL/m^2^)	48.8 ± 22.6) (*n* = 54)	22.9 ± 13.0 (*n* = 107)	< 0.001
LVEDD (*n* = 160) (cm/m^2^)	3.4 ± 0.50 (*n* = 51)	2.7 ± 0.41 (*n* = 109)	< 0.001
SFR (*n* = 165) (n(%))	28 (53%)	2 (2%)	< 0.001

EROA – Effective regurgitant orifice area, LA – Left atrium; LVEDD – Left ventricular end-diastolic dimension; RVol – Regurgitant Volume; SFR – systolic flow reversal

LA and LV are indexed to Body Surface Area

**Fig. 2:**
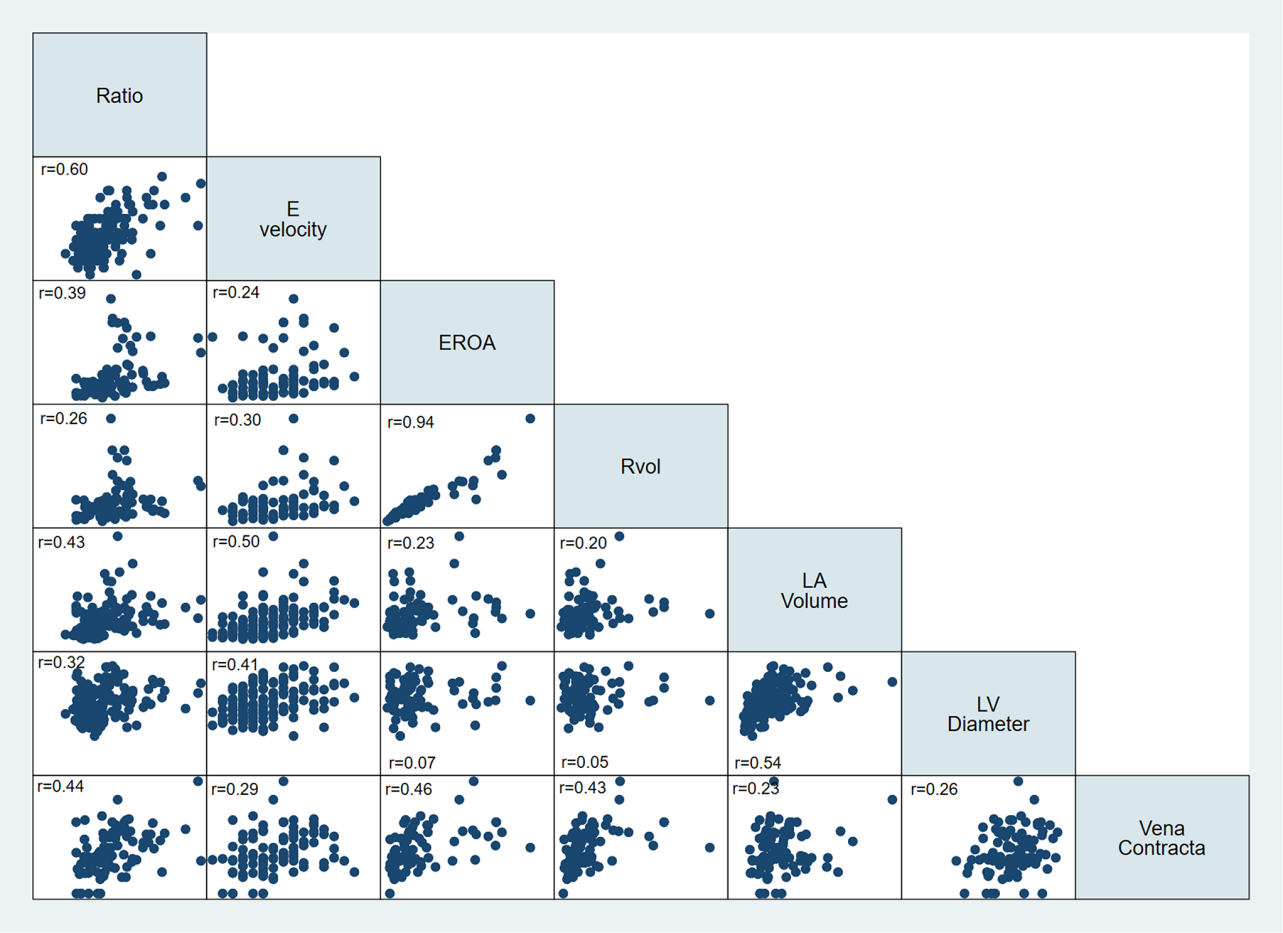
Correlation matrix of qualitative and quantitative MR parameters. Ratio used in this figure is the mitral-aortic VTI ratio

**Fig. 3:**
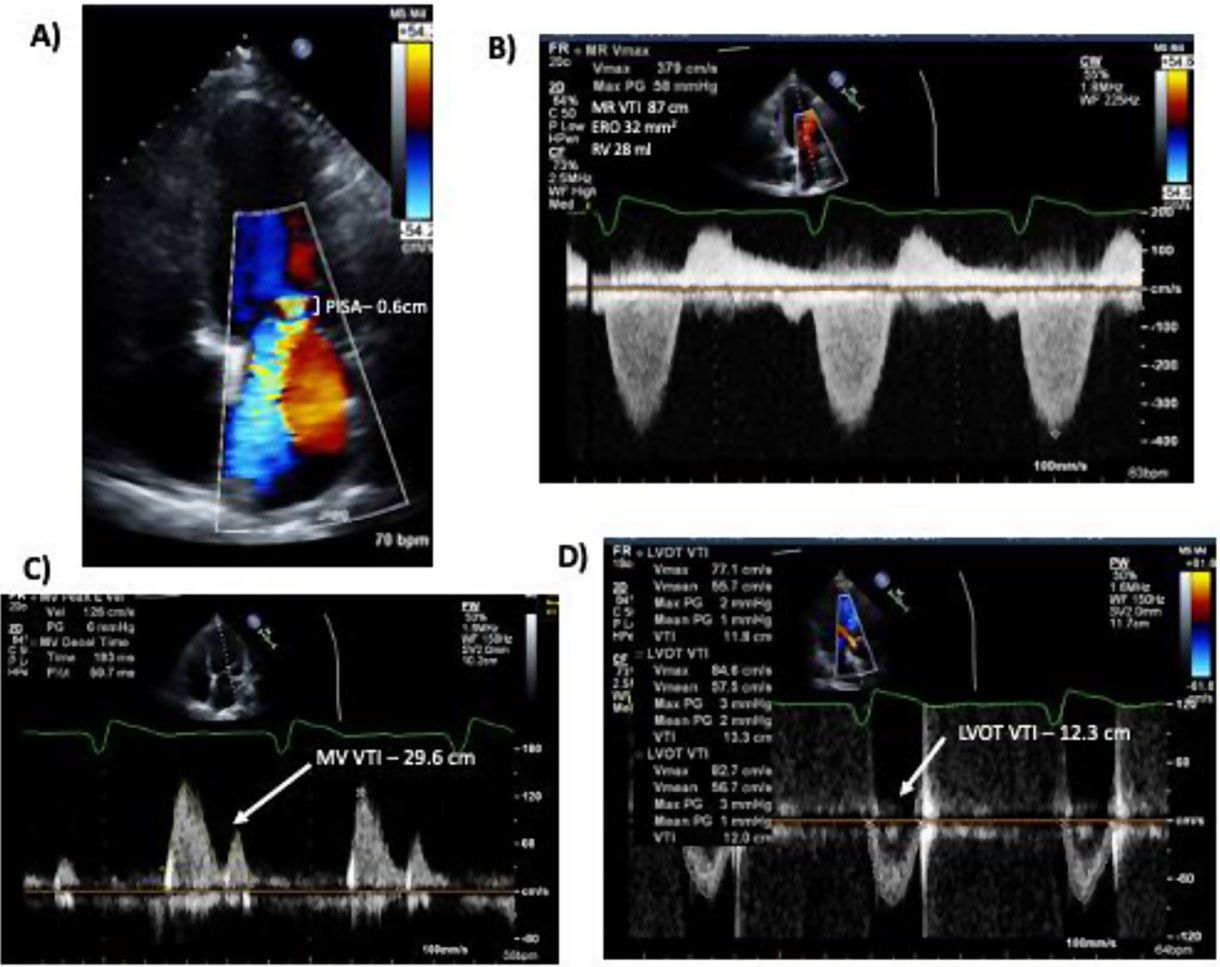
– Example of discordance between mitral-aortic ratio and typical quantitative parameters. An example of a patient with severe mitral regurgitation where typical quantitative parameters may underestimate severity. Panels **A** and **B** show typical quantitative parameters used in mitral regurgitation assessment which are all in the moderate range (PISA 0.6 cm, ERO 32 mm2 and RV 28 ml). The MV VTI: LVOT VTI ratio (panels **C** and **D**) however is high (29.6/12.3 = 2.4) suggestive of severe regurgitation

A correlation matrix between individual MR parameters is demonstrated in Fig. 2. Univariable logistic regression analysis for the association between individual diagnostic parameters and severe MR is demonstrated in Table 2. Increasing mitral-aortic ratio was associated with a near doubling of probability of having severe MR with an AUC of 0.94 (95% CI 0.89–0.97). Using the Youden Index, a calculated optimal threshold of 1.26 for severe MR was identified for the mitral-aortic VTI ratio with a sensitivity of 89%, and specificity of 93%. Analysis with other parameters at their respective recommended thresholds to suggest severe MR was performed and results presented in Table 3. This demonstrates that ratio > 1.3 has a very high diagnostic performance with AUC 0.88. Diagnostic accuracy estimates for Ratio > 1.3 demonstrated a sensitivity of 82%, specificity of 94%, PPV 86%, NPV 91%, an overall accuracy of 90%, a positive likelihood ratio of 13.0 and negative likelihood ratio of 0.20. Estimates for other parameters are described in Table 4. When combining all measurements with the method of DeLong et al., it is still demonstrated that a ratio of > 1.3 results in an AUC of 0.81. An example of use of the mitra-aortic ratio is presented in Fig. 3.

**Table 2:** Association between parameter (continuous) and severe MR

Parameter	Odds Ratio	95% CI	*p*-value	AUC
MV VTI: LVOT VTI Ratio	1.97	1.59–2.43	< 0.001	0.94
MV VTI: RVOT VTI Ratio	1.79	1.48–2.15	< 0.001	0.92
EROA (mm^2^)	1.04	1.01–1.07	0.003	0.76
RVol	1.05	1.02–1.08	0.001	0.77
VC width	1.87	1.39–2.52	< 0.001	0.78
LA Volume	1.11	1.07–1.14	< 0.001	0.88
E-velocity	2.06	1.64–2.58	< 0.001	0.88
LVEDD	5.61	2.97–10.61	< 0.001	0.79

VC – vena contracta. Remaining abbreviations per Table 1

**Table 3:** Association between parameter by threshold and severe MR (univariable)

Parameter	Odds Ratio	95% CI	*p*-value	AUC
MV VTI: LVOT VTI Ratio > 1.3	66.0	23.7-184.5	< 0.001	0.88
MV VTI: RVOT VTI Ratio > 1.14	35.2	12.6–98.7	< 0.001	0.85
EROA > 40 mm	8.5	2.3–31.4	0.001	0.68
RVol > 60%	-			
VC > 7 mm	1.27	0.3–9.6	0.56	0.51
LA dilatation	15.2	6.8–33.6	< 0.001	0.79
LV dilatation	12.5	5.6–27.8	< 0.001	0.75
SFR	61.6	13.8-275.8	< 0.001	0.76
E-Velocity > 1.2	34.1	7.6-153.5	< 0.001	0.68

LA and LV dilatation per ASE recommendations

**Table 4:** Diagnostic performance of parameters for severe MR

Parameter	Sensitivity	Specificity	PPV	NPV	Accuracy
MV VTI: LVOT VTI Ratio > 1.3	82%	94%	86%	91%	90%
MV VTI: RVOT VTI Ratio > 1.14	90%	79%	67%	95%	83%
EROA > 40 mm	45%	91%	89%	53%	64%
RVol > 60%	40%	100%	100%	50%	63%
VC > 7 mm	8%	95%	67%	46%	47%
LA dilatation	74%	84%	70%	87%	81%
LV dilatation	67%	86%	69%	85%	80%
SFR	53%	98%	93%	82%	84%
EVel > 1.2	39%	98%	91%	77%	79%

No difference in AUC was demonstrated with the mitral-aortic VTI ratio for primary vs. secondary MR (0.82 vs. 0.82, *p* = 0.98), and similarly for patients in sinus rhythm vs. non-sinus rhythm (0.89 vs. 0.86, *p* = 0.77). A threshold of > 1.3 reclassified 37% of patients to a diagnosis of severe MR compared to the combination of EROA and vena contracta width.

### Calculation of the mitral-pulmonary ratio as an alternative to the mitral-aortic ratio

**Fig. 4:**
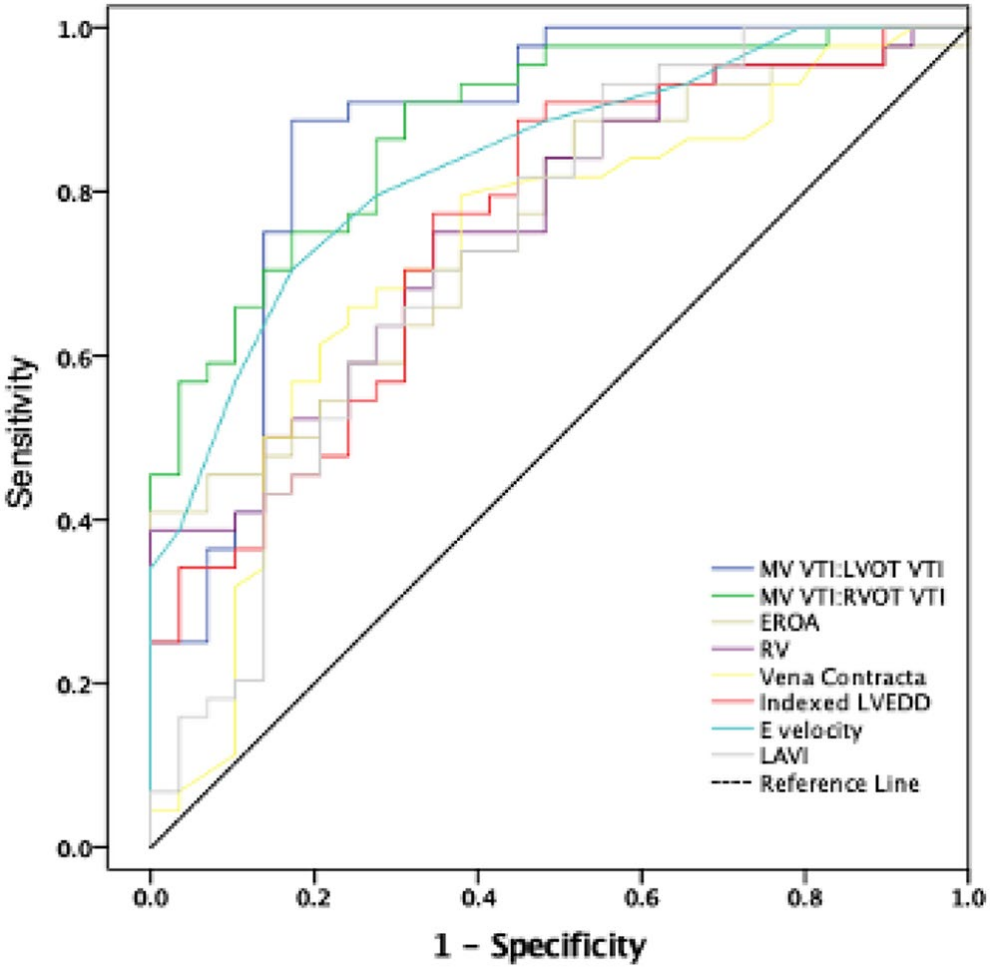
ROC analysis for individual parameters (continuous scale)

**Fig. 5:**
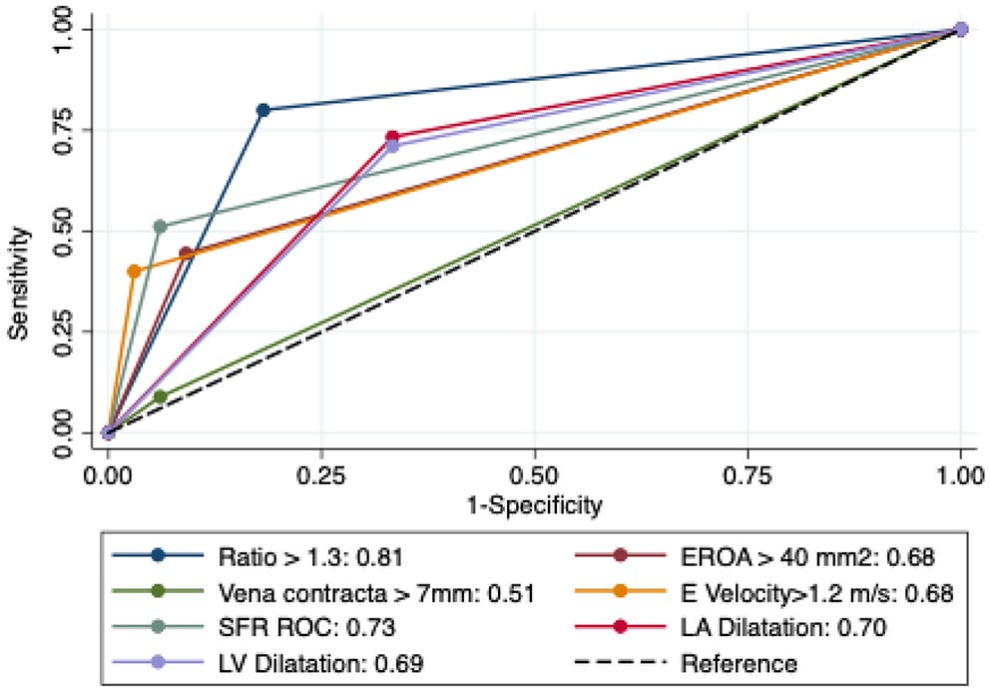
ROC analysis comparing various parameters used in the assessment of severe mitral regurgitation (the mitral: aortic ratio having the largest AUC)

There was a moderate linear relationship between the RVOT CW VTI and LVOT VTI (*r* = 0.71, R^2^ = 0.51). The mitral-pulmonary ratio was increased in patients with severe MR 1.69 ± 0.54 compared with 0.97 ± 0.26 in patients with non-severe MR (*p* < 0.001). The association between the mitral-pulmonary ratio and severe MR was similar to the association noted with the mitral-aortic ratio (OR 1.79 vs. 1.97, AUC 0.92 vs. 0.94). Using the Youden Index, the optimal cut-point for the mitral-pulmonary ratio for severe MR was 1.14 with a sensitivity of 90%, specificity of 79% (AUC 0.85 at cutpoint), correct classification of severe mitral regurgitation grade in 83% of cases, a positive likelihood ratio of 4.4 and negative likelihood ratio of 0.12. When combining all measurements with the method of DeLong et al., a ratio of > 1.14 resulted in an AUC of 0.80. ROC curves for measure of the respective ratios as continous variables, as well as by optimal threshold to indicate severity are presented in Figs. 4 and 5.

### Reproducibility

The mitral-aortic ratio was highly reproducible with inter-observer variability (fellows-in-training vs. expert cardiologists) ICC 0.97 (95% CI 0.94–0.99) and intra-observer variability of 0.98 (95% CI 0.97–0.99).

## Discussion

We have demonstrated that a simple, geometric-free measurement of the ratio between the pulsed wave Doppler VTI of the mitral inflow at the leaflet tips and the LVOT VTI at the aortic annulus is a highly reproducible and accurate metric for severe MR. This marker has a better diagnostic performance than traditional quantitative echocardiographic parameters of vena contracta width, PISA-based EROA and regurgitant volume. A threshold of > 1.3 demonstrated a high sensitivity and specificity for mitral regurgitation and use of this cut off would accurately reclassify 37% of patients to a diagnosis of severe MR compared to the combination of EROA and vena contracta width. We have also demonstrated that the mitral-pulmonary ratio (using the RVOT continuous wave Doppler VTI) in place of the LVOT VTI, produced similar discrimination for severe MR.

Quantitative MR grading methods especially PISA based methods and vena contracta are often regarded as ‘reference standards’ when using echocardiography and are superior to qualitative assessment. However significant limitations of the PISA method make it a less desirable metric. This includes the requirement for a dedicated imaging acquisition with optimisation of the Nyquist limit that cannot be performed in post-processing; inaccuracy when there are multiple jets, eccentric jets or non-circular regurgitant orifice shape [6]; dynamic changes in estimation related to selection of the appropriate timing in the cardiac cycle [7]; magnification of misclassification if small errors in radius measurement due to squaring of this error. Despite all these limitations, EROA and RVol remain highly specific for the diagnosis of severe MR, but their limited sensitivity does not exclude the diagnosis [4]. Often MR jets can be eccentric or there may be multiple jets which makes assessment of severity challenging [3]. Our data demonstrates high specificity for these markers, but their overall accuracy is hampered by suboptimal sensitivity, and an adequate trade-off between these measures of diagnostic performance is needed with diagnostic tests.

The mitral-aortic VTI ratio while subject to errors in tracing and variable sample volume due to translation with the cardiac cycle, may be a useful additional or alternative marker in the presence of some of these PISA limitations. It does not require additional imaging and can be performed in post-processing as it is derived from the core minimum dataset of images required in standard transthoracic echocardiography. It overcomes errors in judgement for maximal velocity by incorporating complete cardiac cycle data and given mitral measurement is in diastole, is not influenced by multiple or eccentric jet morphology. Finally, magnification error is eliminated given the relatively simple formula for calculation that relies on the assumption of proportional mitral and aortic valve area. The measurements are also easily reproducible.

The mitral-aortic VTI ratio is not reliable in clinical situations where the LVOT VTI is increased such as aortic regurgitation or dynamic obstruction. In this situation the substitution of the LVOT VTI with RVOT VTI may be useful and we demonstrated that this produced similar results with a high level of discrimination for severe MR. The use of this ratio has to our knowledge not been described previously in the literature. Pulmonary flow should be equal to aortic flow in the absence of pulmonary regurgitation or intra-cardiac shunts; therefore, the results of our study are consistent with what would be expected physiologically. Although we did not include patients with significant aortic regurgitation or mixed mitral valve disease, the mitral-pulmonary ratio has important applications in these clinical situations. The RVOT continuous wave VTI is measured routinely as part of a standard echocardiogram dataset. Mixed aortic or mitral valve disease is difficult in clinical practice to assess severity and we did not include this group of patients in our study. Further validation of the mitral-pulmonary ratio in a sub-group of patients with mixed mitral valve disease or aortic regurgitation will be required.

Other scenarios where further validation of this metric is required are in patients with multi-valvular pathologies. This parameter also is affected by elevated mitral inflow velocities not attributable to MR. In these instances, assessment of this ratio may be affected by elevation in aortic or mitral velocity-time integrals, which may confound assessment of MR severity. Despite this, we suggest that this metric has a potential utility in other situations in which MR is difficult to assess, such as detecting severe MR post mitral valve intervention. Palmiero et al. demonstrated in a small sample of patients with severe MR that there was a reduction in mean MAVIR from 1.20 to 1.01 [8]. This measure may also have a role in assessment of mitral paravalvular leak post mitral valve replacement, for which echocardiographic assessment is commonly affected by acoustic shadowing.

We noted a small difference in the cut-off for severe MR using the mitral-aortic (1.26) and mitral-pulmonary ratio (1.14). There are two potential reasons for this difference. One potential cause is tricuspid regurgitation. We excluded patients with moderate or greater tricuspid regurgitation. It is likely that mild or mild-moderate tricuspid regurgitation could have contributed to a lower cut-off point noted. Secondly these measures are ratios of mitral valve pulsed-wave Doppler VTI to RVOT/LVOT VTI. These are not equivalent to ratios of stroke volume. Based on the continuity equation, if there are differences noted in LVOT and RVOT diameter, there will be differences in LVOT and RVOT VTI despite the same stroke volume. This could also account for the differences in the VTI ratio noted.

A challenge of mitral regurgitation severity assessment using echocardiography is the high rate of discordance amongst the different parameters underlining the need for an integrated approach. A recent study by Uretsky et al. reported only 8% concordance amongst the ASE recommended parameters with severe MR and although concordance markedly improved with PISA-based EROA, RVol and vena contracta, 38% of patients still had discordant agreement of these parameters [2]. Despite their importance to decision-making, these parameters are reported in less than half of echocardiogram reports by the general medical community. While we do not suggest that the mitral-aortic VTI ratio as a sole marker for use, the ease of its calculation and high diagnostic performance compared to these traditional metrics may improve clinician distinction for severe MR and this feasibility was demonstrated by the high reproducibility in our cohort comparing expert and trainee echo readers.

Few studies have investigated this ratio but have reported similarly high diagnostic accuracy [8–10]. Some of these studies utilised angiographic severity of mitral regurgitation as the reference standard and/or excluded patients in non-sinus rhythm. One study by Afonso et al. in sinus rhythm patients used continuous wave Doppler signal through the mitral inflow. This similarly demonstrated high diagnostic performance with a higher ratio cut-off, however of our method of the VTI ratio to preclude geometric assumptions assumes constant mitral and aortic valve areas and hence we favoured the use of pulse wave Doppler at the mitral leaflet tips which provides assurance of flow at this point only. Similarly, a previous report by Ascione et al. sampled mitral inflow at the annulus and as expected had a lower ratio cut off to predict severe MR. Triboully et al. demonstrated a threshold of 1.3 with sampling at the valve leaflet tips similar to our findings. We build on this initial work by inclusion of a larger sample of severe MR patients, inclusion of consecutive patients irrespective of rhythm, and involvement of trainee echocardiographers to assess the feasibility and ease of this measurement holding an advantage over more complex calculations.

Our study did not utilise cardiac MRI for validation of the VTI ratios; instead the reference standard of expert echocardiographers utilising current echocardiographic guidelines was used. Cardiac MRI to evaluate mitral regurgitation severity is emerging as an important reference standard, and future validation of mitral-aortic VTI ratio with this modality using a prospective study design and a larger number of patients would further strengthen the use of this parameter as an arbiter for severity in echocardiography. The objective of our study was to evaluate a simple and easily accessible parameter to allow rapid disease discrimination. All patients with severe MR were accepted for urgent or emergent intervention by a multi-disciplinary independent Heart Team, with the degree of regurgitation and consequent management being unlikely to change with additional MRI assessment. Cardiac MRI was also not widely available during the study period and is less reliable when there are arrhythmias.

We have also demonstrated the high reproducibility of this metric, not just within observers, but between junior and expert echocardiography readers. Quantitative parameters are limited by significant inter-observer variability [11] which may have implications for treatment decisions and prognosis. The lack of dedicated quantitative measurements as standard requirements in a typical echocardiographic study may also contribute to hesitation in both acquisition and reporting of these metrics with subspecialty imaging cardiologists at best routinely reporting vena contracta width in 75%, EROA 58% and RVol 29% [12]. As mitral tip VTI and LVOT VTI form part of a routine minimum dataset [13], this metric does not require additional clips and can be easily performed in post-processing. The feasibility amongst differing levels of experience is an important advantage over PISA based techniques.

### Limitations

We recognise several significant limitations to our study. Our sample size, while larger than other studies in this area, is still small and larger multi-centre evaluation, with use of MRI as validation will be required to ensure results remain reproducible. We did not evaluate annular changes, or serial changes in measurements which is necessary for test-retest verification as well as usefulness for successive studies and this will be the subject of future study. We did not include patients with mixed mitral valve disease or aortic regurgitation so further validation of the mitral-pulmonary ratio in this group would be required. The association between the mitral-aortic and mitral-pulmonary ratios with remodelling parameters such as LV volume, LV mass and LA volume was not fully investigated in this study. Future research is required to demonstrate that these ratios are independently associated with remodelling parameters.

## Conclusion

The mitral-aortic and mitral-pulmonary VTI ratios are simple, geometric-free parameters feasibly reproducible from routine echocardiographic datasets and is an excellent discriminative tool for severe MR irrespective of MR mechanism or patient rhythm. While we do not suggest that it replace the current paradigm of echocardiographic assessment of MR, the lack of routine clinical acquisition and variable reporting of these typical quantitative parameters suggest that clinicians should consider integration of this ratio in routine reporting.

## Data Availability

No datasets were generated or analysed during the current study.
